# Supplementary material for: Developmental low-dose bisphenol A exposure leads to extensive transcriptome female masculinization and male feminization later in life
Source: Commun Med (Lond). 2025 Oct 1;5:410. doi: 10.1038/s43856-025-01119-8 (PMC12488919; doi:10.1038/s43856-025-01119-8)
Supplement: Supplementary file 2 — Supplementary Information [file 43856_2025_1119_MOESM2_ESM.pdf]

Supplementary Information

Developmental low-dose bisphenol A exposure leads to extensive transcriptome female masculinization and male feminization later in life

Thomas Lind*, Linda Dunder, Margareta H. Lejonklou, P. Monica, Lind, Håkan Melhus, Lars Lind

*Corresponding author: thomas.lind@medsci.uu.se

Content:
Supplementary Figures 1-7
Supplementary Tables 1-6

Supplementary Fig. 1.

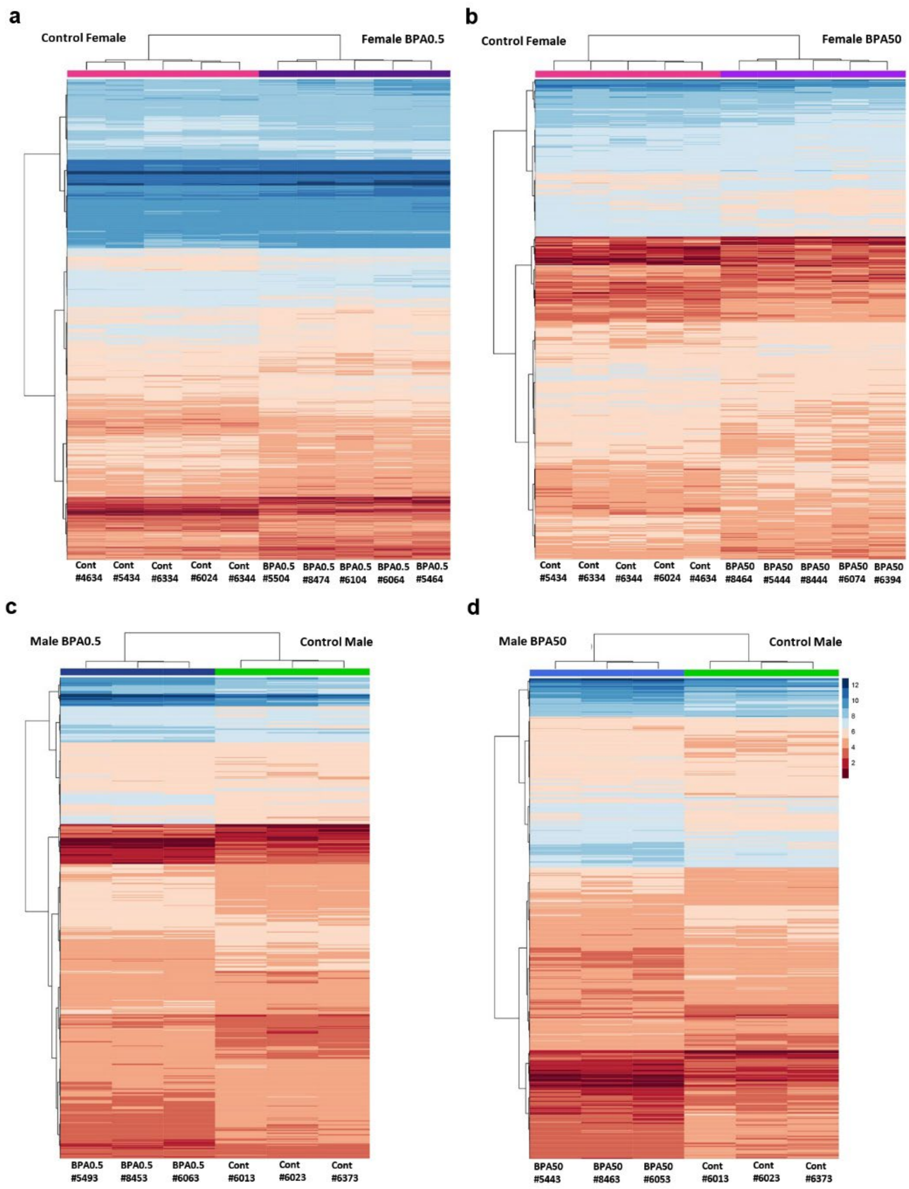

Supplementary Fig. 1. Rat bone marrow transcriptome heatmaps at 52 weeks of age. Heatmaps of microarray differentially expressed genes (DEGs), observed in the bisphenol A (BPA) exposure groups of 0.5 (BPA0.5) or 50 (BPA50) µg BPA/kg BW/day. (**a**) female BPA0.5, (**b**), female BPA50, (**c**), male BPA0.5, (**d**) and male BPA50. Females n=5/group and males n=3/group (Supplementary Data 1). Numbers are individual rat IDs.

Supplementary Fig. 2.

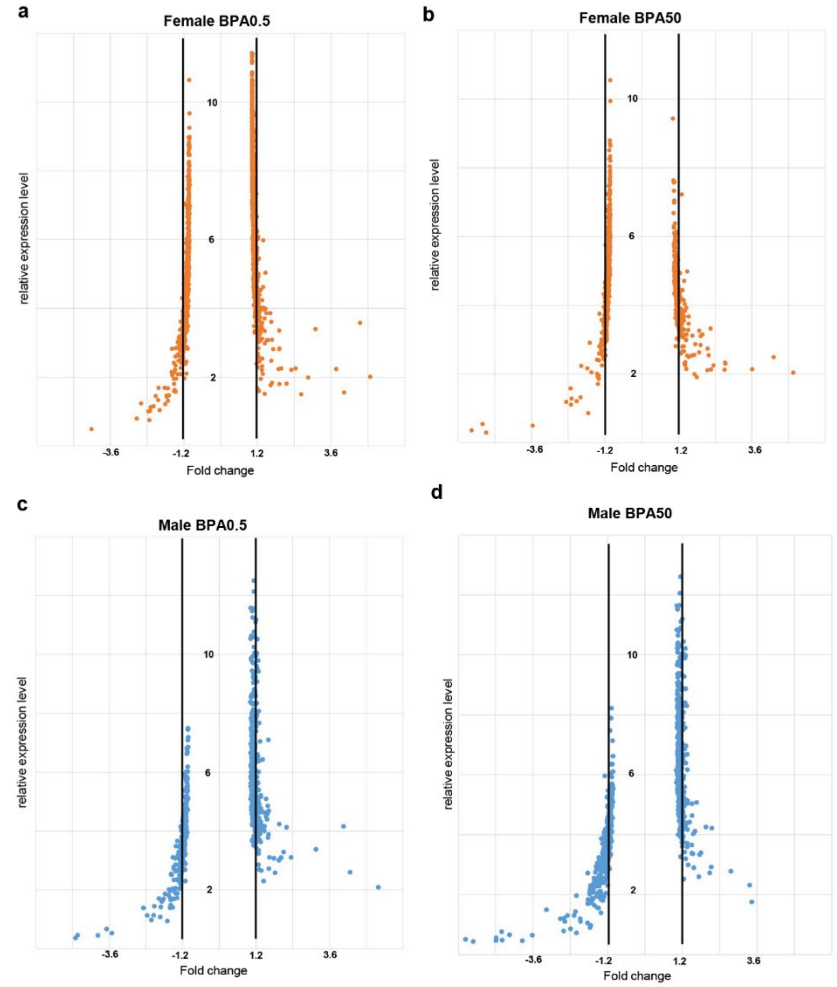

Supplementary Fig. 2. Differentially expressed genes (DEGs) average relative expression level vs fold change (FC). Bisphenol A (BPA)-induced DEGs from exposure groups of 0.5 (BPA0.5) or 50 (BPA50) µg BPA/kg BW/day. (**a**) female BPA0.5, (**b**), female BPA50, (**c**), male BPA0.5, (**d**) and male BPA50 (Supplementary Data 1). The black line highlights a 1.2-fold cutoff. Females n=5/group and males n=3/group.

Supplementary Fig. 3.

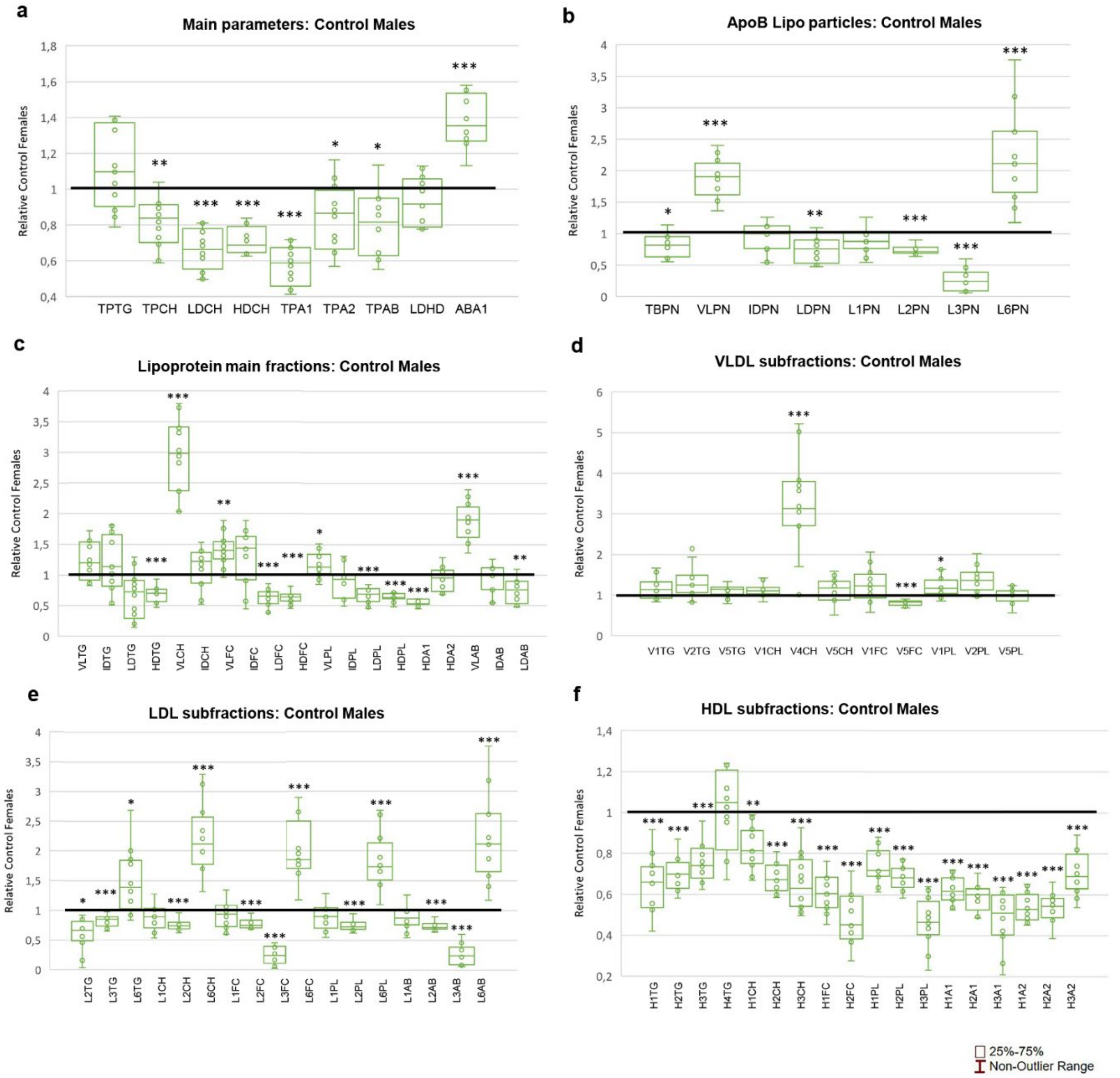

Supplementary Fig. 3. Rat plasma NMR lipoprotein of male controls compared to female controls. (**a**) Main parameters: triglycerides (TPTG), cholesterol (TPCH), LDL-cholesterol (LDCH), HDL-cholesterol (HDCH), Apo-A1 (TPA1), Apo-A2 (TPA2), Apo-B1 (TPAB), LDCH/HDCH (LDHD) and TPAB/TPA1 (ABA1). (**b**) Apo-B particles: Total concentration of Apo-B particles (TBPN), VLDL particle number (VLPN), IDL particle number (IDPN), LDL-1 (1.019-1.030 kg/L) particle number (L1PN), LDL-2 (1.031-1.033 kg/L) particle number (L2PN), LDL-3 (1.034-1.037 kg/L) particle number (L3PN), LDL-6 (1.044-1.063 kg/L) particle number (L6PN). LDL particle densities between 1.037-1.043 (L4PN and L5PN) gave too low signal. (**c**) Lipoprotein main fractions: triglycerides in VLDL (VLTG), triglycerides in IDL (IDTG), triglycerides in LDL (LDTG), triglycerides in HDL (HDTG), cholesterol in VLDL (VLCH), cholesterol in IDL (IDCH), free cholesterol in VLDL (VLFC), free cholesterol in IDL (IDFC), free cholesterol in LDL (LDFC), free cholesterol in HDL (HDFC), phospholipids in VLDL (VLPL), phospholipids in IDL (IDPL), phospholipids in LDL (LDPL), phospholipids in HDL (HDPL), Apo-A1 in HDL (HDA1), Apo-A2 in HDL (HDA2), Apo-B in VLDL (VLAB), Apo-B in IDL (IDAB), Apo-B in LDL (LDAB). Cholesterol in LDL and HDL gave too low signal. (**d**) VLDL 1-5 subfractions (increasing density): triglycerides in VLDL-1 (V1TG), triglycerides in VLDL-2 (V2TG), triglycerides in VLDL-5 (V5TG), cholesterol in VLDL-1 (V1CH), cholesterol in VLDL-4 (V4CH), cholesterol in VLDL-5 (V5CH), free cholesterol in VLDL-1 (V1FC), free cholesterol in VLDL-5 (V5FC), phospholipids in VLDL-1 (V1PL), phospholipids in VLDL-2 (V2PL), phospholipids in VLDL-5 (V5PL). V3TG, V4TG, V2CH, V3CH, V2FC, V3FC, V4FC, V3PL and V4PL gave too

low signal. (**e**) LDL subfractions: triglycerides in LDL-1 (L1TG), triglycerides in LDL-2 (L2TG), triglycerides in LDL-3 (L3TG), triglycerides in LDL-6 (L6TG), cholesterol in LDL-1 (L1CH), cholesterol in LDL-2 (L2CH), cholesterol in LDL-6 (L6CH), free cholesterol in LDL-1 (L1FC), free cholesterol in LDL-2 (L2FC), free cholesterol in LDL-3 (L3FC), free cholesterol in LDL-6 (L6FC), phospholipids in LDL-1 (L1PL), phospholipids in LDL-2 (L2PL), phospholipids in LDL-5 (L5PL), phospholipids in LDL-6 (L6PL), Apo-B in LDL-1 (L1AB), Apo-B in LDL-2 (L2AB), Apo-B in LDL-3 (L3AB), Apo-B in LDL-5 (L5AB), Apo-B in LDL-6 (L6AB). L4TG, L5TG, L3CH, L4CH, L5CH, L4FC, L5FC, L3PL, L4PL and L5AB gave too low signal. (**f**) HDL subfractions: triglycerides in HDL-1 (H1TG) (1.063-1.099 kg/L), triglycerides in HDL-2 (H2TG) (1.100-1.111 kg/L), triglycerides in HDL-3 (H3TG) (1.112-1.124 kg/L), triglycerides in HDL-4 (H4TG) (1.125-1.210 kg/L), cholesterol in HDL-1 (H1CH), cholesterol in HDL-2 (H2CH), cholesterol in HDL-3 (H3CH), free cholesterol in HDL-1 (H1FC), free cholesterol in HDL-2 (H2FC), phospholipids in HDL-1 (H1PL), phospholipids in HDL-2 (H2PL), phospholipids in HDL-3 (H3PL), Apo-A1 in HDL-1 (H1A1), Apo-A1 in HDL-2 (H2A1), Apo-A1 in HDL-3 (H3A1), Apo-A2 in HDL-1 (H1A2), Apo-A2 in HDL-2 (H2A2), Apo-A2 in HDL-3 (H3A2). Results expressed as box plots of control males relative to control females. The black line represents the value of no difference (Supplementary Data 8). Females n=11 and males n=12. *p<0.05, **p<0.01 and ***p<0.001 vs control, Student's t-test.

Supplementary Fig. 4.

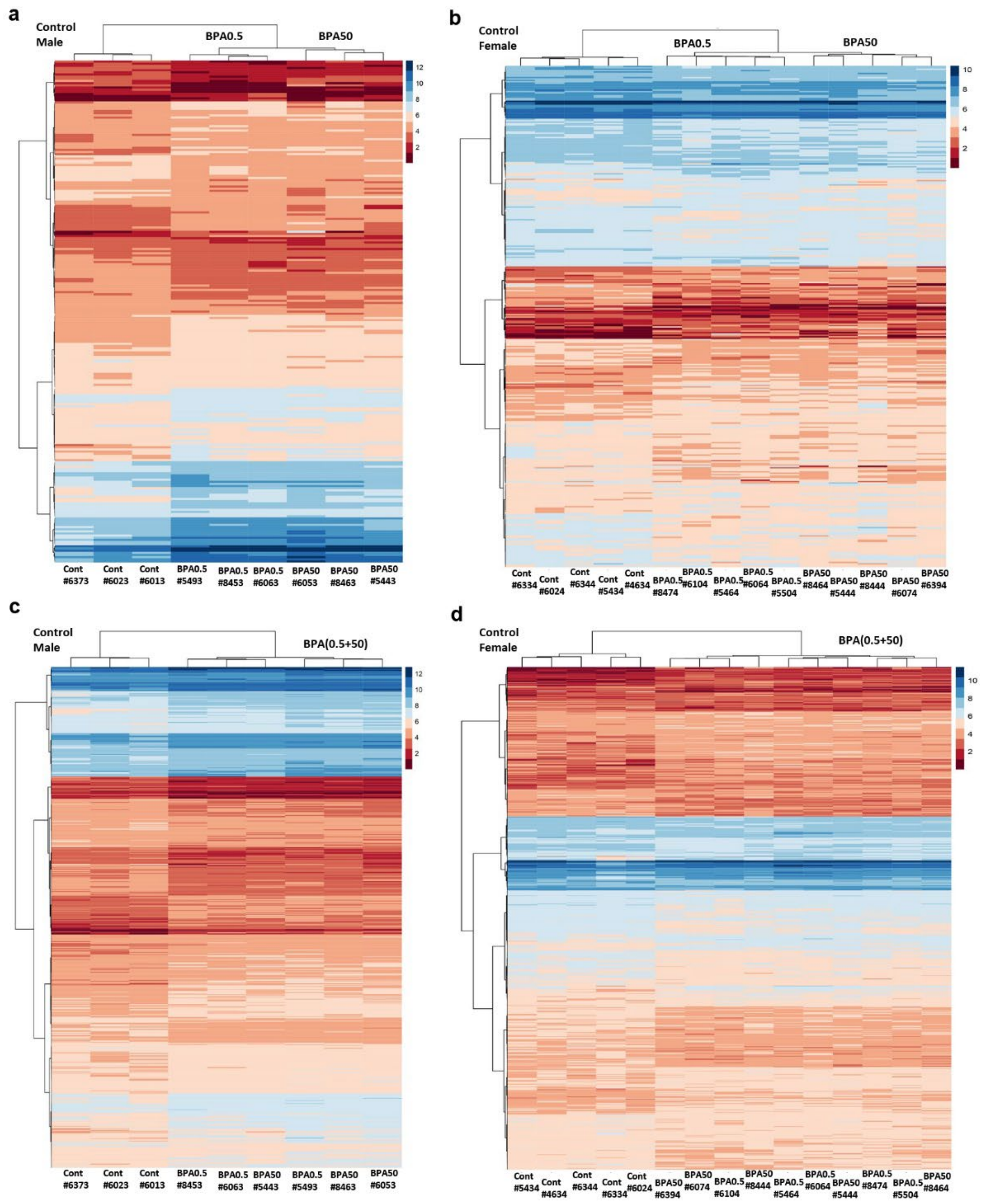

Supplementary Fig. 4. Rat bone marrow transcriptome combined heatmaps at 52 weeks of age exposed to bisphenol A (BPA). Heatmaps of microarray differentially expressed genes (DEGs) presented side by side for male (**a**) and female (**b**) exposed to 0.5 (BPA0.5) and 50 (BPA50) µg BPA/kg BW/day. Females n=5/group and males n=3/group. Heatmaps of combined male BPA(0.5+50) (c) and female BPA(0.5+50) exposure groups (d) (Supplementary Data 1 and 3). Exposed females n=10, exposed males n=6, female controls n=5 and male controls n=3. Numbers are individual rat IDs.

Supplementary Fig. 5.

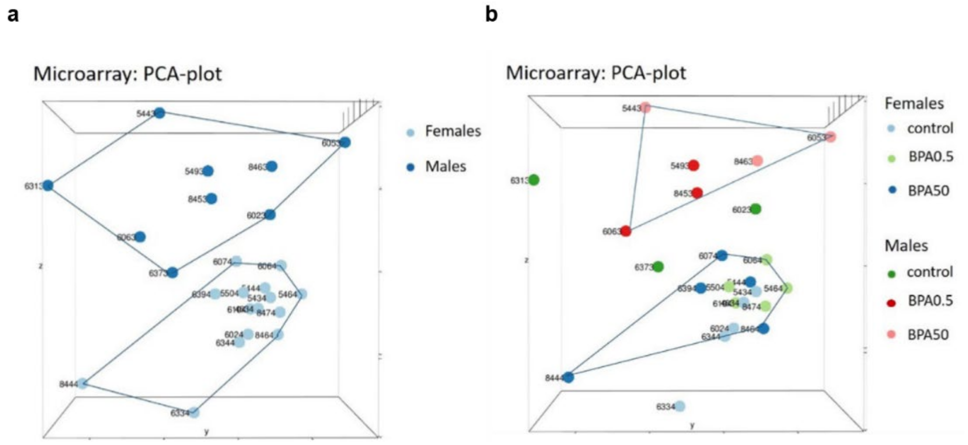

Supplementary Fig. 5. Principal component analysis (PCA) of the bone marrow transcriptome from F344 rat offspring developmentally exposed to 0.5 or 50 µg bisphenol A/kg BW/day. (**a**) PCA plots show grouping of sexes. (**b**) The PCA plot shows the grouping of both bisphenol A doses within each sex (Supplementary Data 1). Numbers are individual rat IDs.

Supplementary Fig. 6.

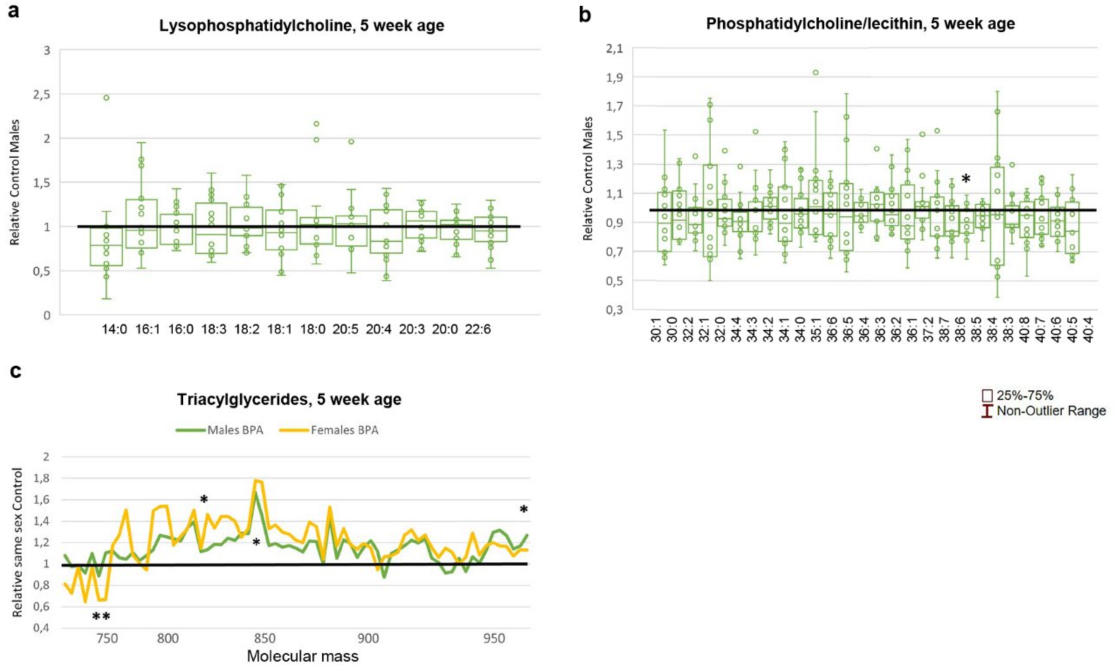

Supplementary Fig. 6. Rat plasma lipidomic profiling in 5-week-old siblings, combined females bisphenol A, BPA(0.5+50) and males BPA(0.5+50) exposure groups. Males (green) (**a**,**b**,**c**) and females (yellow) (**c**). (**a**) Lysophospatidylcholine. (**b**) Phosphatidylcholine/lecithin. (**c**) Triacylglycerides (TG). Results expressed as box plots (or line, c) of exposed females/males relative to female/male controls (Supplementary Data 7). The black line represents the value when there is no difference compared to the same-sex controls. BPA-exposed males n=16, control males n=11, BPA-exposed females n=13, and control females n=11. *p<0.05 and **p<0.01 vs control, Student's t-test.

Supplementary Fig. 7.

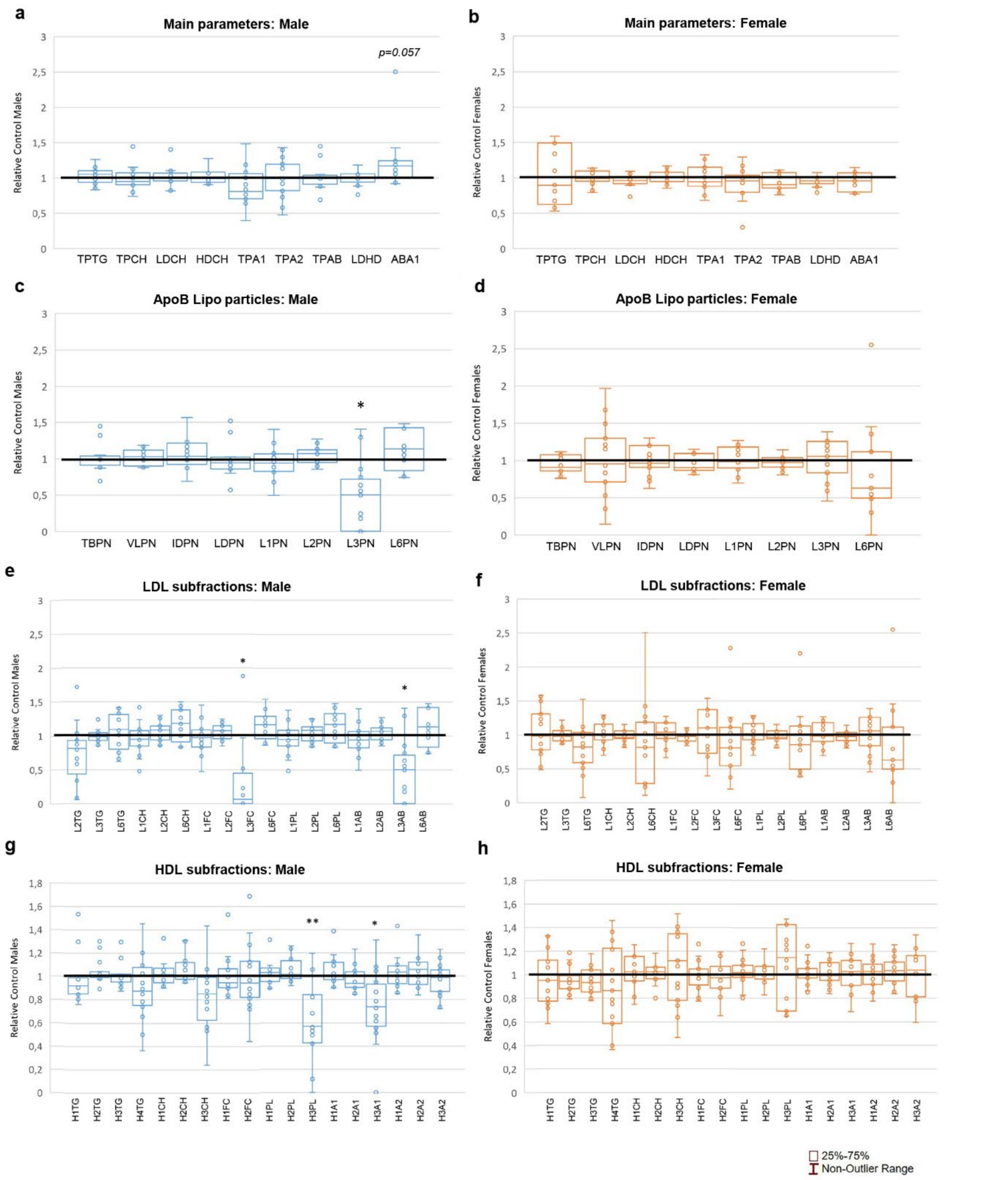

Supplementary Fig. 7. Rat plasma NMR lipoprotein profiling in combined females bisphenol A, BPA(0.5+50) and males BPA(0.5+50) exposure groups. (**a**) Main parameters, males (for explanation see Supplementary Fig. 3). (**b**) Main parameters, females. (**c**) Apo-B particles, males. (**d**) Apo-B particles, females. (**e**) LDL subfractions, males. (**f**) LDL subfractions, females. (**g**) HDL subfractions, males. (**h**) HDL subfractions, females. Results expressed as box plots of exposed females/males relative to female/male controls (Supplementary Data 8). The black line represents the value of no difference compared to the same-sex controls. BPA-exposed males n=16 and control males n=12, BPA-exposed females (n = 15), and control females (n = 11). *p<0.05 and **p<0.01 vs control, Student's t-test.

Supplementary Table 1. The number of shared differentially expressed genes (DEGs) between the different bisphenol A (BPA)-exposed groups and same-sex controls was evaluated to assess sex and dose overlap. Matching shows the number of DEGs regulated in the same direction (up/down) compared to unexposed controls. Opposite shows the number of DEGs regulated (up/down) in the opposite direction compared to unexposed controls. Up represents higher relative expression, and Down represents lower relative expression.

**Group DEG overlap comparison.**	**Total overlap**	**Matching**	**Opposite**	**Up**	**Down**	**Avg. Exp.**
	# (% of group tot)	*# (in %)*	*# (in %)*	*#/#*	*#/#*	*group DEGs*
**Female overlap**						
Female BPA0.5/Female BPA50	279 (16/30%)	235 (84%)	44 (16%)	109/93	170/186	5.36
**Male overlap**						
Male BPA0.5/Male BPA50	230 (27/24%)	204 (87%)	26 (13%)	151/145	79/85	4.96
**BPA0.5 dose overlap**						
Female BPA0.5/Male BPA0.5	150 (9/18%)	69 (46%)	81 (54%)	68/101	82/49	5.38
**BPA50 dose overlap**						
Female BPA50/Male BPA50	110 (12/13%)	39 (35%)	71 (65%)	40/69	70/41	4.93

Avg. Exp. = group total average relative expression level. Control (Cont) Female n=5, Cont Male n=3, BPA0.5 Female n=5, BPA0.5 Male n=3, BPA50 Female n=5 and BPA50 Male n=3.

Supplementary Table 2. As Supplementary Table 1, but with a fold change cutoff of 1.2.

Group DEG overlap comparison	Total overlap *# (% of group tot)*	Same *# (in %)*	Opposite *# (in %)*	Up *#/#*	Down *#/#*	Avg. Exp. *group DEGs*
**Female overlap**						
Female BPA0.5/Female BPA50	34 (17/20%)	31 (91%)	3 (9%)	20/19	14/15	3.01
**Male overlap**						
Male BPA0.5/Male BPA50	64 (25/20%)	53 (83%)	11 (17%)	37/34	27/30	3.63
**BPA0.5 dose overlap**						
Female BPA0.5/Male BPA0.5	17 (9/7%)	9 (53%)	8 (47%)	8/8	9/9	3.32
**BPA50 dose overlap**						
Female BPA50/Male BPA50	14 (11-13%)	4 (29%)	10 (71%)	10/4	4/10	3.31

Avg. Exp. = group total average relative expression level. Control (Cont) Female n=5, Cont Male n=3, BPA0.5 Female n=5, BPA0.5 Male n=3, BPA50 Female n=5 and BPA50 Male n=3.

Supplementary Table 3. The number of shared sex-biased differentially expressed genes (DEGs) and their regulation in the different bisphenol A (BPA)-exposed groups. Matching shows the number of DEGs regulated in the same direction (up/down) compared to female controls over male controls (FC/MC), i.e., female regulation. Opposite shows the number of DEGs regulated (up/down) in the opposite direction compared to FC/MC, i.e., male regulation.

**DEG overlap comparison with sex-biased DEGs (FC/MC)**	**Total overlap** *# (% of group tot)*	**Matching** *# (in %)*	**Opposite** *# (in %)*	**Up** *# (in %)*	**Down** *# (in %)*
Female BPA0.5/FC	429 (24%)	91 (21%)	338 (79%)	150 (35%)	279 (65%)
Female BPA50/FC	347 (38%)	41 (12%)	306 (88%)	102 (29%)	245 (71%)
Male BPA0.5/MC	307 (36%)	263 (86%)	44 (14%)	212 (69%)	95 (31%)
Male BPA50/MC	313 (32%)	279 (89%)	34 (11%)	215 (69%)	98 (31%)

Control (Cont) Female n=5, Cont Male n=3, BPA0.5 Female n=5, BPA0.5 Male n=3, BPA50 Female n=5 and BPA50 Male n=3.

Supplementary Table 4. Top results from Ingenuity Pathway Analysis (Qiagen.com) of combined groups of developmentally exposed female bisphenol A, BPA(0.5+50) and male BPA(0.5+50) rats.

**Female BPA(0.5+50)**	**description**	**p-value**
Disease	Cancer	*2.1E-2 – 3.4E-8*
	Organism injury and abnormalities	*2.1E-2 – 3.4E-8*
Clinical chemistry and hematology	Increased levels of potassium	*3.8E-4 – 3.8E-4*
	Increased levels of CRP	*4.2E-2 – 4.2E-2*

**Male BPA(0.5+50)**		
Disease	Inflammatory response	*7.0E-3 – 1.3E-7*
	Endocrine system disorders	*5.0E-3 – 6.4E-7*
Clinical chemistry and hematology	Increased levels of AST	*1.5E-1 – 5.6E-3*
	Increased levels of ALT	*1.5E-1 – 1.7E-2*

Control (Cont) Female n=5, Cont Male n=3, BPA0.5+50 Female n=10, BPA0.5+50 Male n=6. CRP (C-reactive protein), AST (aspartate aminotransferase), ALT (alanine aminotransferase). Right-tailed Fisher's exact test

Supplementary Table 5. Human MetS NMR metabolomics, sex-specific changes (Supplementary Data

4.

Metabolite name	p-value M	beta M	beta F	p-value F
Very-large HDL cholesterol esters (%)	0,0900908	-0,1364	-0,4619	2,04E-14
Small VLDL cholesterol esters (%)	0,8713823	-0,01	0,3389	2,52E-06
Very-large HDL triglycerides	0,2765577	0,0683	-0,2974	4,29E-06
Medium VLDL cholesterol esters	0,2068834	0,0836	0,2885	0,0000119
LDL-cholesterol	0,1194023	0,1017	0,2582	0,0001106
Omega 3 fatty acids (%)	0,2440756	-0,0732	-0,2558	0,0002222
Large LDL cholesterol esters	0,3383242	0,0626	0,2419	0,0002969
Very small VLDL cholesterol	0,5718874	0,0367	0,2231	0,0005256
Large LDL total concentration	0,4721398	0,0473	0,2205	0,0008946
Large VLDL triglycerides (%)	0,4605852	0,0455	-0,2274	0,0020646
Small LDL cholesterol (%)	0	-0,8108	-0,1059	0,09477
Large LDL cholesterol (%)	1,88E-08	-0,3624	-0,0312	0,638437
IDL cholesterol	2,23E-07	-0,3363	-0,1177	0,0740391
IDL cholesterol esters	5,99E-07	-0,3245	-0,1089	0,0993768
Total phophoglycerides	9,52E-07	0,3185	0,0403	0,5132957
22:6, Docosahexsaenoic acid	9,44E-06	0,2859	0,0655	0,3219862
Large LDL free cholesterol	9,68E-06	-0,2903	-0,0649	0,3304098
IDL phospholipids	0,0000244	-0,2776	0,01	0,8766697
Small VLDL cholesterol (%)	0,0000342	-0,2552	-0,0311	0,6667319
Phosphatidylcholine	0,0000748	0,2571	0,0143	0,8177308

M=Male and F=Female, logistic regression analysis, beta coefficient=beta.

Supplementary Table 6. Human MetS lipidomics, sex-specific changes (Supplementary Data 5).

Chemical name	p-value M	beta M	beta F	p-value F
N6-carbamoylthreonyladenosine	0,1804565	0,0826	0,3885	1,80E-08
3-(3-amino-3-carboxypropyl)uridine	0,1097458	0,0975	0,3865	2,72E-08
C-glycosyltryptophan	0,2455059	0,0688	0,3566	6,48E-08
cystine	0,7597132	0,0194	0,3673	6,52E-08
andro steroid monosulfate C19H28O6S (1)	0,5984022	0,0316	0,3069	5,53E-06
dihomo-linolenoylcarnitine (C20:3n3 or 6)	0,7019808	0,0251	0,3041	7,16E-06
1-(1-enyl-stearoyl)-2-arachidonoyl-GPE (P-18:0/20:4)	0,234889	-0,0791	-0,3148	0,000012
leucylglycine	0,621969	-0,0277	-0,2785	0,000036
5,6-dihydrouridine	0,9728218	0,0021	0,2853	0,000058
4-oxo-retinoic acid	0,36691	-0,0595	-0,2774	0,000071
2-hydroxy-3-methylvalerate	1,03E-07	0,2986	0,1111	0,103186
1,5-anhydroglucitol (1,5-AG)	1,82E-07	-0,3606	0,0877	0,164965
etiocholanolone glucuronide	2,83E-07	-0,3266	-0,0681	0,322826
N-acetyltryptophan	3,07E-07	0,3193	0,1083	0,140281
glutamine_degradant	7,96E-07	-0,2598	0,0291	0,607082
4-methyl-2-oxopentanoate	1,17E-06	0,265	0,0797	0,191626
deoxycholic acid glucuronide	1,55E-06	0,3129	0,0599	0,390031
octadecenedioylcarnitine (C18:1-DC)	3,82E-06	-0,3043	-0,0392	0,570474
pregnenediol sulfate (C21H34O5S)	3,91E-06	-0,2553	-0,019	0,766883
kynurenate	0,0000102	0,2823	0,1235	0,081586

M=Male and F=Female, logistic regression analysis, beta coefficient=beta.
