# Supplementary material for: Developmental low-dose bisphenol A exposure leads to extensive transcriptome female masculinization and male feminization later in life
Source: Commun Med (Lond). 2025 Oct 1;5:410. doi: 10.1038/s43856-025-01119-8 (PMC12488919; doi:10.1038/s43856-025-01119-8)
Supplement: Supplementary file 3 — Description of Additional Supplementary Files [file 43856_2025_1119_MOESM3_ESM.pdf]

Description of Additional Supplementary files

File name: Supplementary Data 1

Description: The complete DEG lists from each exposure group

File name: Supplementary Data 2

Description: Gene set enrichment analysis using Enrichr

File name: Supplementary Data 3

Description: The DEG list from combined exposure groups

File name: Supplementary Data 4

Description: Plasma NMR metabolomics of MetS in women and men

File name: Supplementary Data 5

Description: Plasma lipidomics of MetS in women and men

File name: Supplementary Data 6

Description: Rat plasma ELISA and enzymatic assays

File name: Supplementary Data 7

Description: Rat plasma Lipidomics

File name: Supplementary Data 8

Description: Rat plasma NMR metabolomics
