# Supplementary material for: Developmental low-dose bisphenol A exposure leads to extensive transcriptome female masculinization and male feminization later in life
Source: Commun Med (Lond). 2025 Oct 1;5:410. doi: 10.1038/s43856-025-01119-8 (PMC12488919; doi:10.1038/s43856-025-01119-8)
Supplement: Supplementary file 12 — reporting summary [file 43856_2025_1119_MOESM12_ESM.pdf]

nature portfolio

Corresponding author(s): Thomas Lind

Last updated by author(s): Aug 11, 2025

Reporting Summary

Nature Portfolio wishes to improve the reproducibility of the work that we publish. This form provides structure for consistency and transparency in reporting. For further information on Nature Portfolio policies, see our Editorial Policies and the Editorial Policy Checklist.

Statistics

For all statistical analyses, confirm that the following items are present in the figure legend, table legend, main text, or Methods section.

n/a	Confirmed	
☐	☒	The exact sample size (*n*) for each experimental group/condition, given as a discrete number and unit of measurement
☐	☒	A statement on whether measurements were taken from distinct samples or whether the same sample was measured repeatedly
☐	☒	The statistical test(s) used AND whether they are one- or two-sided<br>*Only common tests should be described solely by name; describe more complex techniques in the Methods section.*
☐	☒	A description of all covariates tested
☐	☒	A description of any assumptions or corrections, such as tests of normality and adjustment for multiple comparisons
☐	☒	A full description of the statistical parameters including central tendency (e.g. means) or other basic estimates (e.g. regression coefficient) AND variation (e.g. standard deviation) or associated estimates of uncertainty (e.g. confidence intervals)
☐	☒	For null hypothesis testing, the test statistic (e.g. *F*, *t*, *r*) with confidence intervals, effect sizes, degrees of freedom and *P* value noted<br>*Give P values as exact values whenever suitable.*
☒	☐	For Bayesian analysis, information on the choice of priors and Markov chain Monte Carlo settings
☒	☐	For hierarchical and complex designs, identification of the appropriate level for tests and full reporting of outcomes
☒	☐	Estimates of effect sizes (e.g. Cohen's *d*, Pearson's *r*), indicating how they were calculated

Our web collection on statistics for biologists contains articles on many of the points above.

Software and code

Policy information about availability of computer code

Data collection	no software was used
Data analysis	STATA16.1 was used for calulations of the human data

For manuscripts utilizing custom algorithms or software that are central to the research but not yet described in published literature, software must be made available to editors and reviewers. We strongly encourage code deposition in a community repository (e.g. GitHub). See the Nature Portfolio guidelines for submitting code & software for further information.

Data

Policy information about availability of data

All manuscripts must include a data availability statement. This statement should provide the following information, where applicable:

- Accession codes, unique identifiers, or web links for publicly available datasets
- A description of any restrictions on data availability
- For clinical datasets or third party data, please ensure that the statement adheres to our policy

The microarray data discussed in this publication will be deposited in Annotare – EMBL-EBI (https://www.ebi.ac.uk › annotare) and are accessible through number XXX when accepted for publication.
The Swedish law does not permit us to make human health data available in the public domain. However, the data could be obtained for research purposes upon a reasonable request from the authors.

Human research participants

Policy information about studies involving human research participants and Sex and Gender in Research.

Reporting on sex and gender	All analyses were sex-stratified, since previous studies have indicated differernt effects of BPA in males and femals
Population characteristics	The basic characteristics of the human cohort has been given.
Recruitment	This has been described. Random recruitment from the population that hopefully would not lead to any bias.
Ethics oversight	The Swedish Ethics Board.

Note that full information on the approval of the study protocol must also be provided in the manuscript.

Field-specific reporting

Please select the one below that is the best fit for your research. If you are not sure, read the appropriate sections before making your selection.

☒ Life sciences ☐ Behavioural & social sciences ☐ Ecological, evolutionary & environmental sciences

For a reference copy of the document with all sections, see nature.com/documents/nr-reporting-summary-flat.pdf

Life sciences study design

All studies must disclose on these points even when the disclosure is negative.

Sample size	The animal study was n=8-12/group, a sufficient numbers for bone tissue and blood analysis in our experience. For the main analysis, the microarray, n=5/group for females and n=3/group for males, more samples in female groups as their individual variation is larger than males, from experience.
Data exclusions	No data exclusion for the main analysis, microarray (n=24 samples) and lipidomics (tot n=56 samples). From NMR, tot n=55 samples, excluded n=1, due to extreme values. Extreme values were excluded from boxplots of enzymatic kits, multiplex, and metabolites (NMR). Not predefined.
Replication	Experiments were performed using well established methods.
Randomization	Rats were randomly divided into 3 groups/sex, pups of the same sex and dosing group all had different mothers.
Blinding	Main analyses (microarray, lipidomics and NMR analysis) were performed at blinded at dedicated facilities.

Reporting for specific materials, systems and methods

We require information from authors about some types of materials, experimental systems and methods used in many studies. Here, indicate whether each material, system or method listed is relevant to your study. If you are not sure if a list item applies to your research, read the appropriate section before selecting a response.

Materials & experimental systems

n/a	Involved in the study	
☐	☒	Antibodies
☒	☐	Eukaryotic cell lines
☒	☐	Palaeontology and archaeology
☐	☒	Animals and other organisms
☐	☒	Clinical data
☒	☐	Dual use research of concern

Methods

n/a	Involved in the study	
☒	☐	ChIP-seq
☒	☐	Flow cytometry
☒	☐	MRI-based neuroimaging

Antibodies

Antibodies used	Meso Scale multiplex assay kits: K153ACL, K15179C and K15095D
Validation	Kit components validated by company.

Animals and other research organisms

Policy information about studies involving animals; ARRIVE guidelines recommended for reporting animal research, and Sex and Gender in Research

Laboratory animals	F344/DuCrl rats, 52 week old
Wild animals	study did not involve wild animals
Reporting on sex	Sex-based analysis is an important par of this work
Field-collected samples	study did not involve samples collected from the field
Ethics oversight	The animal study was carried out at an Uppsala University animal facility. Ethical approval (C26:13) was given by the Ethical Committee on Animal Research, and the study followed guidelines laid down by the European Union Legislation (Convention ETS123 and Directive 2010/63/EU).

Note that full information on the approval of the study protocol must also be provided in the manuscript.

Clinical data

Policy information about clinical studies

All manuscripts should comply with the ICMJE guidelines for publication of clinical research and a completed CONSORT checklist must be included with all submissions.

Clinical trial registration	The human part is a population-based epidemiological study without intervention and is therefore not reported as a clinical trial
Study protocol	The protocol has been given as a reference.
Data collection	This has been described in the methods section.
Outcomes	Outcome is the metabolic syndrome. Exposures are a great set of metabolites.
